# A simple preparation protocol for shipping and storage of tissue sections for laser ablation-inductively coupled plasma-mass spectrometry imaging

**DOI:** 10.1093/mtomcs/mfac013

**Published:** 2022-03-16

**Authors:** Rebecca Buchholz, Sebastian Krossa, Maria K Andersen, Michael Holtkamp, Michael Sperling, Uwe Karst, May-Britt Tessem

**Affiliations:** Institute of Inorganic and Analytical Chemistry, University of Münster, Münster, Germany; Department of Circulation and Medical Imaging, Norwegian University of Science and Technology, Trondheim, Norway; Department of Circulation and Medical Imaging, Norwegian University of Science and Technology, Trondheim, Norway; Institute of Inorganic and Analytical Chemistry, University of Münster, Münster, Germany; Institute of Inorganic and Analytical Chemistry, University of Münster, Münster, Germany; European Virtual Institute for Speciation Analysis (EVISA), Münster, Germany; Institute of Inorganic and Analytical Chemistry, University of Münster, Münster, Germany; Department of Circulation and Medical Imaging, Norwegian University of Science and Technology, Trondheim, Norway; Clinic of Surgery, St. Olavs Hospital, Trondheim University Hospital, Trondheim, Norway

**Keywords:** LA–ICP–MSI, tissue preparation, human prostate, zinc

## Abstract

A rapid and cost-efficient tissue preparation protocol for laser ablation-inductively coupled plasma-mass spectrometry imaging (LA–ICP–MSI) has been developed within this study as an alternative to the current gold standard using fresh-frozen samples or other preparation techniques such as formalin fixation (FFix) and formalin-fixed paraffin-embedding (FFPE). Samples were vacuum dried at room temperature (RT) and stored in sealed vacuum containers for storage and shipping between collaborating parties. We compared our new protocol to established methods using prostate tissue sections investigating typical endogenous elements such as zinc, iron, and phosphorous with LA–ICP–MSI. The new protocol yielded comparable imaging results as fresh-frozen sections. FFPE sections were also tested due to the wide use and availability of FFPE tissue. However, the FFPE protocol and the FFix alone led to massive washout of the target elements on the sections tested in this work. Therefore, our new protocol presents an easy and rapid alternative for tissue preservation with comparable results to fresh-frozen sections for LA–ICP–MSI. It overcomes washout risks of commonly used tissue fixation techniques and does not require expensive and potentially unstable and time-critical shipping of frozen material on dry ice. Additionally, this protocol is likely applicable for several bioimaging approaches, as the dry condition may act comparable to other dehydrating fixatives, such as acetone or methanol, preventing degradation while avoiding washout effects.

## Graphical Abstract

**Graphical Abstract:**
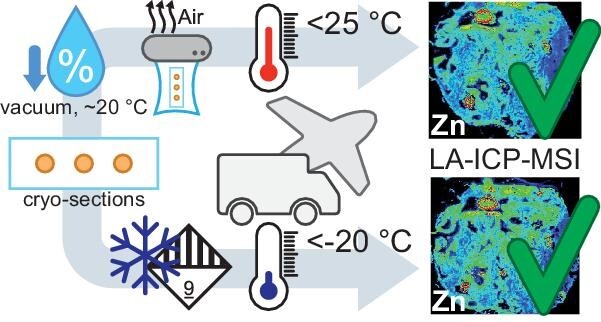
Vacuum dried and sealed samples result in equivalent data quality as fresh frozen samples using LA-ICP-MSI.

## Introduction

Interdisciplinary research projects, where groups of experts need to work together across international borders, are becoming increasingly common and provide significant contributions to science. Since the different collaboration partners in such projects are often located not only in other institutions, but also in other cities or even countries, extra attention must be given to methods of preservation, storage, and transfer to ensure sample integrity. For bioimaging approaches of tissue sections, mainly three different types of sample preservation are commonly used: cryo/fresh frozen, formalin-fixed frozen, and formalin-fixed paraffin-embedded samples (FFPE).^1^ Tissue samples prepared and preserved according to FFPE protocols are commonly used in histology and pathology due to their high-quality conservation of morphological integrity and almost unlimited storability under ambient conditions.^2^ In contrast, in detection of various biological molecules, such as metabolites, RNA, and DNA, fresh-frozen tissue is considered to be the gold standard.

For molecular imaging experiments using matrix-assisted laser desorption/ionization mass spectrometry imaging (MALDI–MSI), the problem of tissue integrity and analyte accessibility, especially using FFPE tissue, is well known and discussed.^1^ For example, FFPE tissue requires paraffin removal and antigen retrieval to allow imaging of proteins. This can negatively affect the composition and spatial distribution of proteins.^3^ FFPE tissues are easily available and are thus, despite these disadvantages, widely used for MALDI–MS imaging of tryptic peptides in various sample types such as pancreatic and breast cancer,^4^ ovarian cancer,^5^ and prostate cancer.^6^

For analysis of molecules such as metabolites and lipids, fresh-frozen tissue is preferred over FFPE tissue, as it requires no or only brief tissue wash, limiting analyte delocalization and washout.^7^ However, methods for metabolite and lipid detection in either formalin-fixed or FFPE tissue have been developed.^7,8^ Paraffin embedding and subsequent dewaxing lead to severe washout effects, whereas using only the formalin fixation (FFix) step on its own, yielded similar results to fresh-frozen samples in mouse kidney for lipid imaging.^8^ Using a carefully designed washing procedure, metabolites were detected in FFPE tissue using MALDI–MSI and allowed differentiation between normal and tumor tissue.^7^

The effects of sample preparation protocols on the suitability of different tissues for the analysis by laser ablation-inductively coupled plasma (LA–ICP) MSI have barely been investigated. As LA–ICP–MSI is an element-specific imaging technique and does not distinguish between different binding forms of the analysed elements, absolute quantification is obtained. As mentioned earlier, molecules such as lipids, metabolites, endogenous peptides, and proteins are affected differently by washout effects during sample preparation. This applies also to metal species. Earlier studies by Bischoff *et al.*^9^ and Olynyk *et al.*^10^ already compared fresh samples against FFPE samples, but both studies were performed as bulk analysis after acid digestion and not for imaging purposes. Hare *et al.* reported nearly complete loss of K and Mg and moderate loss of Fe, Cu, and Zn after paraformaldehyde fixation and sucrose cryoprotection of mouse brain for bulk analysis with ICP–MS^11^ and suggested relevance for LA–ICP–MSI.^12^ Bonta *et al.* addressed the problem of sample preparation for LA–ICP–MSI in the brain, liver, and heart tissue and found that the alkali metals Na, K and the alkaline earth metal Mg were extremely affected by the washout effect in FFPE compared to fresh-frozen tissue. For the transition metals, Mn and Ni, the washout effect was less prominent. They also reported contamination (for Ca and Zn) during sample preparation, which has also to be taken into account when working with FFPE tissue.^13^ A comparison of freeze drying and oven drying of chicken liver for bulk ICP–MS and LA–ICP–MS analysis concluded that both methods may alter the elemental composition of soft tissue. Notably, freeze drying resulted in clear reduction of Mg, Ca, Mn, Cu, Zn, Sr, P, and S while Na, Cl, and K levels were enhanced in LA–ICP–MS tissue surface analysis.^14^

Accurate determination of zinc in prostate tissue is of high relevance for prostate cancer research, as zinc is a promising diagnostic biomarker. In contrast to other tissue types, the healthy prostate contains exceptionally high (2–4 µmol/g wet weight) zinc concentrations necessary to facilitate the high citrate levels secreted by the prostate. In malignant prostates, zinc concentration is significantly lower (about 50–80% reduction) than that of healthy prostate.^15,16^ Additionally, because of this exceptionally high zinc content in normal prostate tissue, analysing zinc concentration is well suited to indicate, even small, potential washout effects caused by various sample handling and preparation procedures.

In this study, we developed a new sample preparation protocol involving drying at RT followed by vacuum packaging. This approach overcomes the disadvantages of commonly used methods, either requiring elaborative storage and transport in frozen conditions or fixation protocols causing washout of target elements. To check the suitability of this protocol for LA–ICP–MSI analysis, we compared our new protocol with conventional sample preparations protocols such as FFix and paraffin embedding.

## Materials and methods

### Materials

If not stated otherwise, all reagents were obtained from Sigma-Aldrich (St. Louis, MO, USA), VWR (Radnor, PA, USA), or Carl Roth (Karlsruhe, Germany).

### Sample collection and preparation

This study was approved by the Regional Committee for Medical and Health Research Ethics of Central Norway (identifier 2017/576) and all procedures followed national and EU ethical regulations. All patient donors signed an informed written consent before a whole-mount 2 mm prostate tissue slice was collected from the middle of the prostate as soon as possible after prostatectomy. The tissue slice was snap frozen and stored in Biobank 1 (St. Olavs Hospital, Trondheim, Norway) at −80°C. The procedure is described in detail by Bertilsson *et al.*^17^ For this study, the donor was anonymized and one circular (3 mm diameter) tissue sample was drilled from the peripheral zone, while keeping the tissue frozen. Four 10 µm cryo-sections of the drilled sample were stained with hematoxylin, eosin, and saffron (HES) and scanned before a pathologist from St. Olavs Hospital confirmed that the sample only contained normal, non-malignant tissue.

**Fig. 1:**
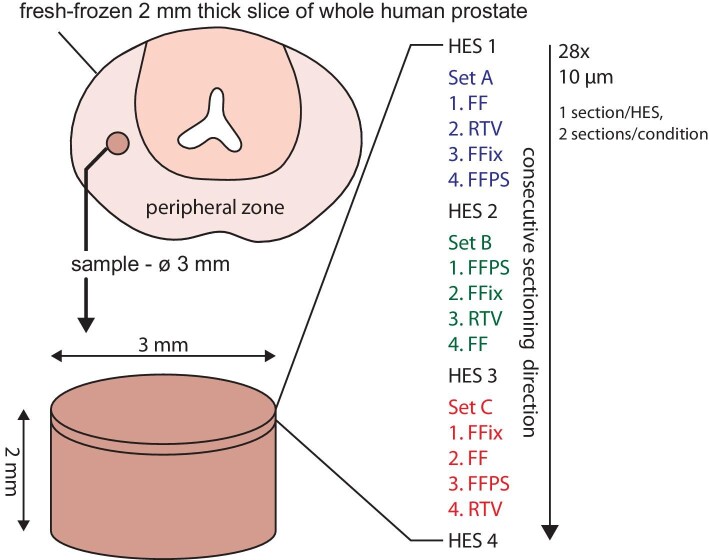
Schematic overview of the prostate tissue sample collection and sectioning. The cylindric sample (3 mm diameter, 2 mm height) was collected from the peripheral zone of a slice of whole-mount human prostate and subsequently cryo-sectioned. The consecutive sectioning order of the three replicates (sets A, B, and C) with randomized section order is shown for each protocol per set (FF = fresh frozen, RTV = room temperature vacuum dried and sealed, FFix = formalin fixed, FFPS = formalin fixed, paraffin sealed).

For LA–ICP–MSI, 10 µm thick consecutively cut cryo-sections were mounted onto regular light microscopy glass slides (two sections per slide). The slides were prepared immediately after sectioning with four different protocols and storage conditions (three technical replicates, shuffled order, see Fig. 1):

***Protocol 1-FF*** (fresh frozen): Sections were placed in microscope slide mailer cases and stored at −80°C until shipment on dry ice in an expanded polystyrene (EPS) container.

***Protocol 2-RTV*** (room temperature vacuum dried and sealed): Sections were dried for 15 min at RT under vacuum and stored in vacuum-packed microscope 5-slide mailer cases at 4°C until shipment in a cooled EPS container.

***Protocol 3*** *-* ***FFix*** (formalin fixed): Sections were fixed and washed [15 min, 4% formaldehyde in PBS, 30 s ddH_2_O, 30 s 70% (v/v) ethanol], dried for 20 min at RT under vacuum and stored in vacuum-packed microscope slide mailer cases at 4°C in until shipment in a cooled EPS container.

***Protocol 4*** *-* ***FFPS*** (formalin fixed, paraffin sealed): Sections were fixed and washed (15 min, 4% formaldehyde pH 7, 30 s ddH_2_O), dehydrated [1× tap water, 1 × 80% (v/v) ethanol, 1 × 96% (v/v) ethanol, 3× absolute ethanol; 3x Tissue-Tek Tissue-Clear, Sakura Finetek, CA, USA] using the Tissue-Tek Prisma (Sakura Finetek) according to manufacturer’s recommendations, sealed with paraffin, and stored at RT until shipment in a cooled EPS container.

Vacuum for drying and packing was applied by first using a desiccator connected to a consumer-grade vacuum pump for 15 min (reaching ∼450 hPa after 50 s), followed by vacuum packing using a consumer-grade food vacuum sealer (reaching ∼300 hPa after 30 s). All sections were packed into the same EPS container to guarantee same transport time and environmental effects during shipment. The FF sections were embedded in dry ice at the bottom of the EPS container followed by a layer of EPS and paper to thermally isolate them from the RTV, FFix, and FFPS sections on top to avoid refreezing and to ensure stable temperatures below 25°C during transport. The transportation time from Trondheim, Norway, to Münster, Germany, was approximately 48 h. The time from sample packaging until measurement was approximately 3 months.

Sections from protocols 1, 2, and 3 were used without further preparation. For protocol 4, the paraffin was removed in a xylene bath for approximately 10 min, until it was removed completely. Afterwards the sections were rehydrated in decreasing ethanol baths (100, 90, and 70% EtOH) and water for 2 min each.

### External calibration based on matrix-matched standards

Quantification of zinc, the element of interest, was carried out using matrix-matched standards based on gelatin. For preparation of a stock solution (c[Zn] = 10 mg mL^–1^) ZnCl_2_ (Alfa Aesar, Karlsruhe, Germany) was dissolved in doubly distilled water, and standard solutions ranging from 1 to 500 µg mL^–1^, were prepared by serial dilution. Respectively, 100 mg of gelatin (Grüssing GmbH, Filsum, Germany) were spiked with 900 µL of the standard solution, resulting in six gelatin-based standards ranging from 0.9 to 450 µg·g^–1^ and a blank (no zinc added). For homogenization, all standards were heated up to 60°C and vortexed until the gelatin was completely dissolved.

Gelatin standards were cryo-sectioned into 10-µm-thin sections and mounted onto glass slides. For external calibration, 10 lines per standard were ablated with parameters described in the following section, intensities were averaged and applied against the zinc concentration.

To validate the zinc concentration in the gelatin standards, 50 mg of each standard were digested with 1 mL of concentrated nitric acid (Merck KGaA, Darmstadt, Germany). The solutions were filled up to 50 mL and subsequently diluted to a zinc concentration between 5 and 30 ng·L^–1^. Calibration standards were prepared by diluting a zinc ICP-Standard in 2% HNO_3_ (v/v). All standards, calibration, and digested gelatin standards were analysed via liquid introduction ICP–MS (ICPMS 2030, Shimadzu, Kyoto, Japan). The ICP–MS was equipped with Ni sampler and skimmer cones, a quartz injector pipe and a low Ar consumption mini-torch.

### Bioimaging by means of LA–ICP–MSI

For elemental bioimaging, the previously described ICP–MSI was coupled via tygon tubing to a LSX 213 G2 + laser ablation system (Teledyne Cetac, Thousand Oaks, CA, USA). The microscopic slides were placed into a 2-volume HelEx ablation cell and the tissue sections and gelatin standards were ablated in a line-by-line scan and the created aerosol was transported via He flow (0.5 L·min^–1^ cell gas and 0.3 L·min^–1^ cup gas) into the plasma torch. Additional 0.45 L·min^–1^ Ar flow was added after the ablation cell. As a compromise between spatial resolution and analysis time, 15 µm spot size and 45 µm·s^–1^ scan speed was used. Laser energy was optimized for quantitative ablation.

Transient signals for the two zinc isotopes ^64^Zn and ^66^Zn with dwell times of 75 ms and the isotopes ^31^P and ^57^Fe with dwell times of 50 ms were recorded. Signals were converted into 2D images with an in-house developed software (ImaJar, written by Robin Schmid, University of Münster). The same software was used to quantify zinc data and to evaluate averaged concentrations or signal intensities over the tissue section.

Applying the 3- and 10-σ criteria, limit of detection and limit of quantification were determined as 2.7 and 8.8 µg·g^–1^, respectively, for the spot size of 15 µm. The averaged signals obtained for the calibration standards [relative standard deviations (RSDs) between 2.5 and 9%] across the whole concentration range could be sufficiently fitted to a linear function with a correlation coefficient *R*2 of 0.996 or better.

To determine the average concentration within the tissue, pixels with no relation to the tissue were excluded. For this approach, pixels with low phosphorous intensity (offset: <1100 cps) were excluded from averaging. Averaging of the hotspot regions was performed by defining the region of interest with freehand drawing. To assess washout effects the determined concentrations were calculated into recovery rates, with the FF sections set to 100%.

### Segmentation of LA–ICP–MSI data for zinc distribution analysis

For quantification and comparison of zinc distribution between different tissue regions, the MS images were segmented by using the ^31^P signal to first identify on-tissue pixels (vs non-tissue) and then to secondly segment the on-tissue pixels into stroma, epithelial glands, and glandular lumen pixels. All classification thresholds were empirically determined, and the resulting segmentation was verified by comparison with the morphology of the respective brightfield images and adjacent HES-stained sections. For pixel classification of non-tissue, tissue, and lumen, the ^31^P signal contrast was increased by clipping the data to the 0.25–0.50 quantile interval. The resulting data were transformed into 8-bit grayscale images, followed by Gaussian blurring (5 × 5 px kernel), Otsu thresholding, and contour detection using the respective functions from OpenCV.^18^ Pixels enclosed by the contour with the largest area were set as tissue. Contours inside the “tissue contour” were filtered by ^31^P signal to obtain lumen: contours that enclosed pixels with a median ^31^P signal smaller than the 0.45 quantile and were surrounded (area obtained by morphologically dilating the respective contour by a 5 × 5 px kernel) by pixels with a median ^31^P signal larger than the 0.55 quantile of the full ^31^P dataset were considered as glandular lumen. Due to the high ^31^P concentration difference, glands and stroma were segmented by using the on-tissue ^31^P data: the data were first clipped to the 0.05–0.95 quantile interval and transformed to 8-bit grayscale images. These were segmented by a combination of Gaussian blurring (5 × 5 px kernel), Otsu thresholding, morphological opening (2 × 2 px kernel, 1 iteration), and contour detection using the respective functions from OpenCV. ^18^ Contours were filtered by size (contours <0.05% image size were rejected). Pixels on or inside the found contours with a ^31^P signal larger than the median were classified as high ^31^P (gland) otherwise as low ^31^P (stroma). Pixels were annotated as “prostate stones” when the pixels were luminal and had a high ^64^Zn content. We defined a pixel as high ^64^Zn (after clipping the raw data to the 0.01–0.99 quantile range) when: $^{64}Z{n_{\rm pixel}} > 0.75\,\,{\rm quantile} ( {^{64}Z{n_{0.01 - 0.99}}} ) + 1.5 * IQR( {^{64}Z{n_{0.01 - 0.99}}} )$. Prior to quantitative analysis, all data/images were cropped to rectangles of 100 × 150 px (1500 × 2250 µm) representing approximately the same area of the consecutive cut tissue sections to improve comparability and avoid problematic regions at the tissue edge containing folded tissue. The complete code and raw data to perform this analysis and image generation is openly available (see section data availability for details).

## Results and discussion

### Bioimaging by LA–ICP–MSI in human prostate tissue

**Fig. 2:**
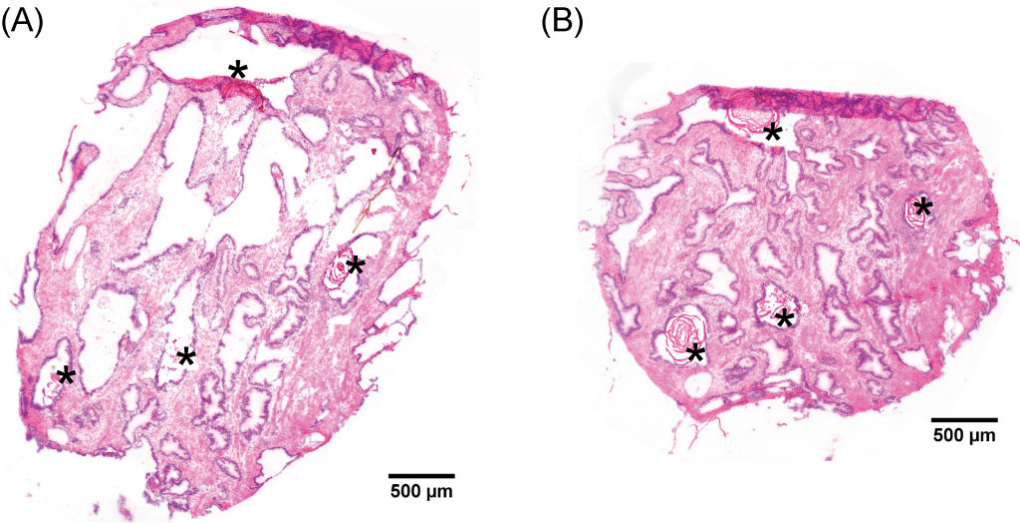
Histology of the prostate tissue sample. Bright-field microscopic images of the two hematoxylin, eosin, and saffron (HES) stained prostate tissue sections adjacent to replicate set A (A: HES 1, B: HES 2 as indicated in Fig. 1). Corpora amylacea/small prostatic calculi are marked with asterisks.

To assess the morphology and histology of the used cylindrical (3 mm diameter) human prostate tissue sample, thin sections at regular intervals (Fig. 1) were HES stained and evaluated by a trained pathologist, who found no signs of cancerous glands (Fig. 2). The chosen sample consisted of roughly 40% gland epithelium and 60% stroma tissue and contained several *corpora amylacea* or small *prostatic calculi* with diameters ranging from 150 to 400 µm (indicated with asterisks in Fig. 2).

To quantify and evaluate the effect of tissue preparation and storage, we recorded the distribution of the essential elements phosphorous, zinc, and iron by LA–ICP–MSI after applying the four different protocols in three replicates. We compared the four different tissue preparation protocols of (i) FF, (ii) RTV, (iii) FFix, and (iv) FFPS (Fig. 1), and since zinc was the target element in this study, calibration was based on matrix-matched gelatin zinc standards.

**Fig. 3:**
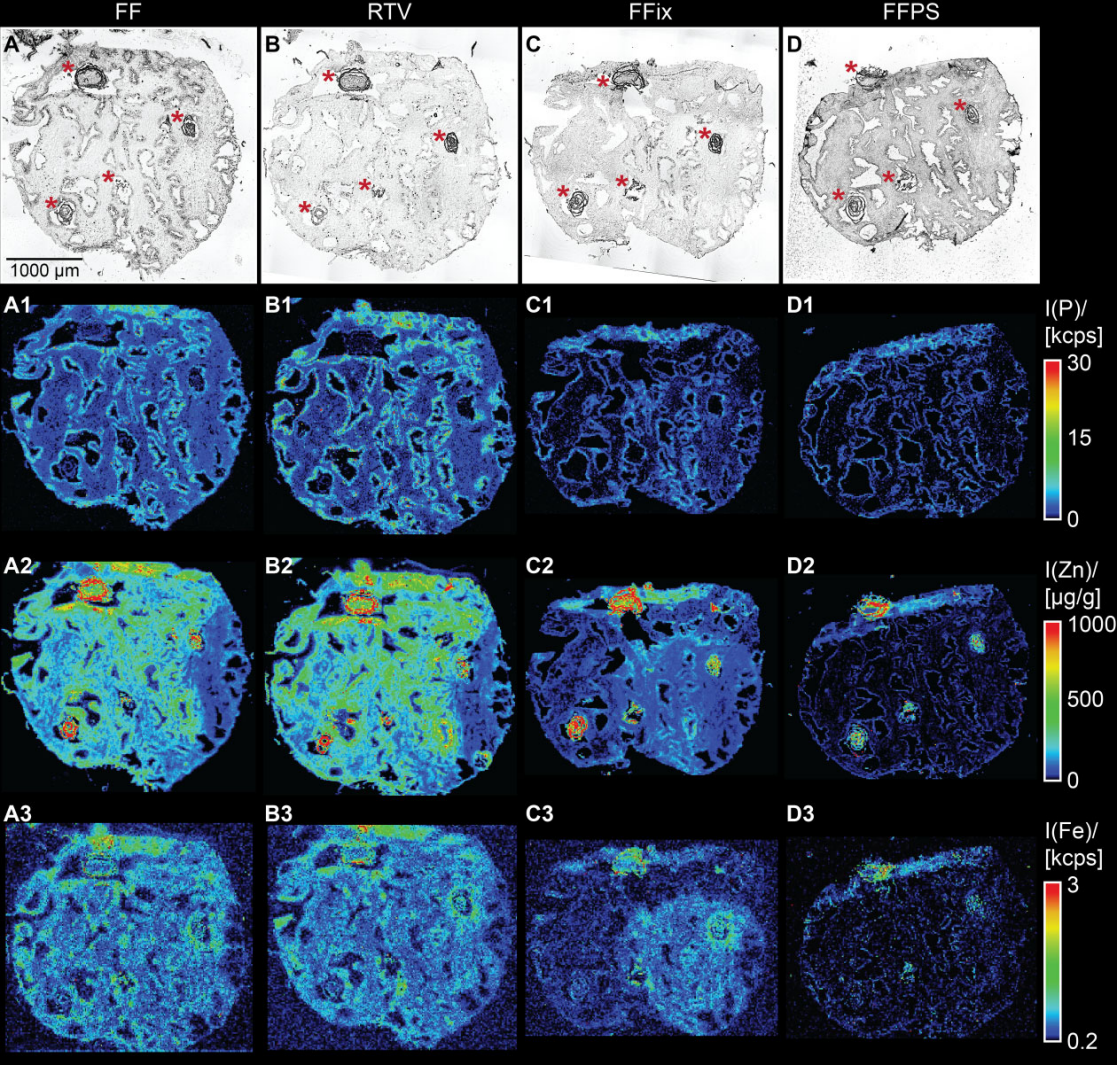
Phosphorous, zinc, and iron distributions after using the four different tissue preparation and storage protocols 1–4 presented in Fig. 1 (replicate set A). Shown are bright-field microscopic images (AD) and pseudo color images of the qualitative phosphorous (A1–D1), iron (A3–D3), and quantitative zinc (A2–D2) distributions as obtained by laser ablation-inductively coupled plasma-mass spectrometry imaging (LA–ICP–MSI). Images are of serial sections of the same tissue sample, which were preserved, stored, and shipped according to the FF (A), RTV (B), FFix (C), and FFPS (D) protocols. Calibration for zinc was performed using matrix-matched standards based on gelatin. Zinc hotspot areas are marked with asterisks in the bright-field microscopic images. (FF = fresh frozen, RTV = room temperature vacuum dried and sealed, FFix = formalin fixed, FFPS = formalin fixed, paraffin sealed).

Zinc isotopes ^64^Zn and ^66^Zn were both detected and showed high spatial correlation, excluding interferences on either isotope. Therefore, only ^64^Zn was used for further evaluation. Bioimages for all four protocols of replicate set A (Fig. 1) for phosphorous, zinc, and iron are shown in Fig. 3. Phosphorous distribution (E–H) demonstrated a homogenous background with higher phosphorous intensity in the glandular epithelium surrounding tissue free glandular lumen and was in good accordance with the microscopic image (A–D).

Zinc was in general less homogenously distributed in all four protocols compared to for example phosphorous. average zinc concentration was approximately 300 µg·g^–1^ in the tissue, whereas there were also zinc hotspots aligning with the *corpora amylacea* and *prostatic calculi* (compare Figs. 1 and 2, marked in the microscopic images with asterisks) with concentrations over 1000 µg·g^–1^ (average 600–700 µg·g^–1^). For the sake of conciseness, we will refer to these areas as zinc hotspots in the tissue from this point on.

To recognize significant “washout effects” from the different preparation protocols, the intersectional variation was determined between the three sample sets (sets A–C, Fig. 1) as the RSD between parallel thin sections. Therefore, average intensities for each element within each section of the three fresh-frozen sections were compared to each other. The intersectional variation was 1.5% for phosphorous, 6.8% for zinc, and 3.2% for iron. These alterations can be explained both by biological variation within the tissue sample and by shearing and distortions from cryo-sectioning as illustrated in Fig. 1. Since phosphorous intensities were overall quite homogenously distributed, the intersectional variation between the sample sets was low when comparing the samples sets for all the investigated elements zinc, and iron. The zinc hotspots with very high local concentrations led to higher RSD since the composition in these structures varied significantly from one section to the adjacent, both due to true biological changes and sectioning artifacts. For example, analysing the zinc variability of these hotspots individually in the fresh-frozen samples resulted in RSDs up to 102%.

### Significant wash out of zinc during sample preparation

Alterations in the zinc distributions for the different protocols can already be observed at first glance on the images (Fig. 3). Whereas zinc concentrations and distribution for frozen and dried (FF and RTV) sections were similar, the difference was already severe when introducing a relatively short fixation step with minimal washing steps (FFix sections) and was even worse when the sections were also paraffin sealed (FFPS sections).

**Fig. 4:**
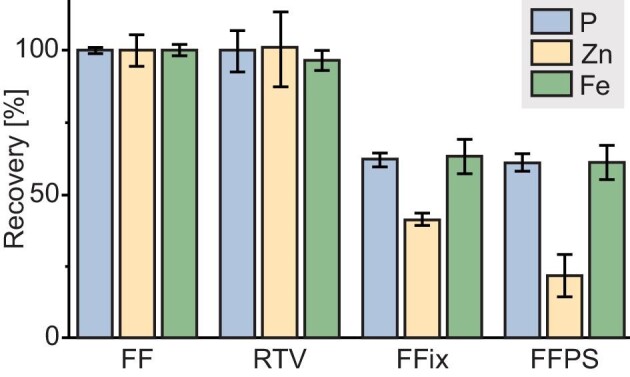
Recovery rates of the four tested protocols. Shown are box plots of the recovery rates obtained (*n* = 3 technical replicates) over the whole sections for the elements phosphorous, zinc, and iron of RTV, FFix, and FFPS relative to FF. (FF = fresh frozen, RTV = room temperature vacuum dried and sealed, FFix = formalin fixed, FFPS = formalin fixed, paraffin sealed).

Zinc as well as the other tested elements (phosphorous and iron) showed relative recovery rates for the dry (RTV) condition compared to the frozen (FF) condition of ≥96.2% (Fig. 4). Thus, drying and vacuum storage led to no relevant loss of material. This is in good accordance with what was expected since none of the compounds are volatile, nor did we observe alterations of the tissue morphology that would indicate damage or loss of material.

After fixation and subsequent washing steps with doubly distilled water and ethanol (FFix), the recovery rates for all elements tested were reduced. The highest reduction was observed for zinc. Its concentration in the overall tissue already decreased to 41% compared to that of the frozen (FF) section. When paraffination and deparaffination were introduced as preparation steps, the average recovery rate reached only 22% for zinc. This means that in this set of sections, nearly 80% of the target analyte were lost during sample preparation. This is similar to Bonta *et al.*, who also observed reduced recovery rates of several elements in FFPE compared to fresh-frozen tissue sections after measuring with LA–ICP–MSI.^13^

### Sub-structures within the tissue are differentially affected by preparation method

**Fig. 5:**
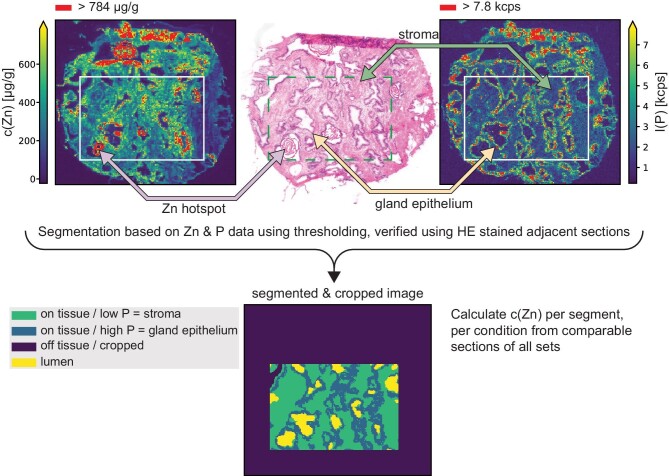
Segmentation of MSI data was based on zinc (top left) and phosphorous (top right) data by thresholding and verification using adjacent hematoxylin, eosin, and saffron (HES) stained sections (top middle, features indicated by arrows). As position and morphology slightly changed between the consecutive sections, all MS images were cropped to equally sized smaller images covering roughly the same area of the original sample to increase comparability (indicated with white boxes on Zn and P distribution images and as a dashed green box on the HES image). Cropped images were segmented based on the P contrast and distribution into on tissue/low P = stroma, on tissue/high P = gland epithelium, lumen, and off tissue.

**Fig. 6:**
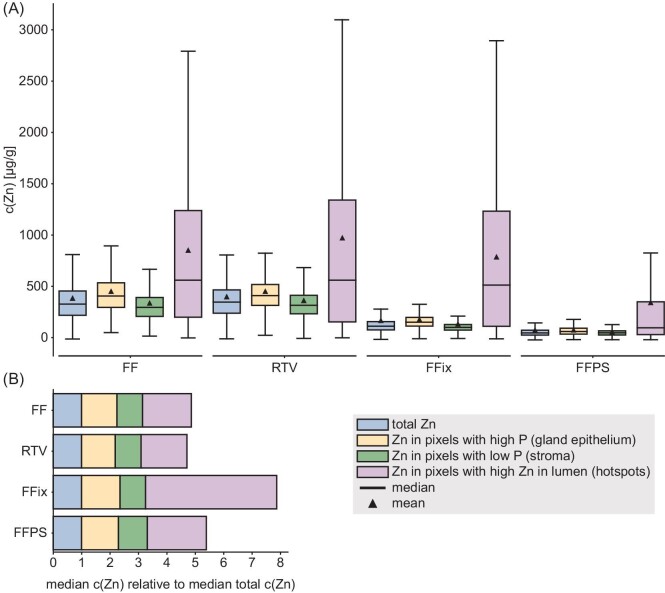
Analysis of the zinc content in gland epithelium, stroma, and hotspots for all four protocols. (A) Box-and-whisker plot [box: Q1 to Q3 quartile, whisker: 1.5 * IQR (IQR = Q3 − Q1) from edges of the box] of the zinc concentration distribution obtained from image segmentation and pixel classification as indicated in Fig. 5 for each protocol. (B) The relative distributions of zinc between gland epithelium, stroma, and hotspots for each protocol (normalized to respective protocol's total zinc) are shown as stacked boxplots. (FF = fresh frozen, RTV = room temperature vacuum dried and sealed, FFix = formalin fixed, FFPS = formalin fixed, paraffin sealed).

LA–ICP–MSI analysis revealed that zinc in the hotspot regions (Figs. 2 and 3, marked with asterisks) appeared unaffected by the washout effect of the FFix protocol while visual inspection indicates otherwise for stroma and gland epithelium (Fig. 3). Motivated by this observation of the apparent differences in the magnitude of the zinc washout between the hotspot regions and the rest of the tissue and the much stronger overall washout of zinc as compared to phosphorous and iron, we wondered whether the washout is also different for the prostate epithelial gland and stroma. In contrast to the hotspot regions, such potential differences were not directly visible from the images alone (Fig. 3). Comparing the morphology of adjacent HES-stained sections with the phosphorous images (Fig. 5) and the brightfield images (Fig. 3), it was apparent that pixels with a high phosphorous content originate mostly from gland epithelium while low phosphorous indicates stroma. This contrast was also unaffected by the washout (Fig. 3A1–D1). This is supported by the fact that the cell density in the gland epithelium is significantly higher than in the stroma, consequently resulting in higher concentrations of phosphorous containing molecules, such as for instance DNA or phospholipids, in the gland epithelium. The relative strong contrast between high and low phosphorous pixels appeared to be stable under all protocols independently of the absolute phosphorous counts. Thus, we chose to use this relative contrast in combination with the high zinc contrast between hotspots and the rest of the tissue as basis to classify every pixel as either most likely gland epithelium, stroma, or hotspot (Fig. 5). After segmentation the respective zinc concentrations were calculated separately for each class and sample preparation protocol using data from all sections (Fig. 6). Considering the large number of pixels per class and protocol (on average 20 022 for stroma, 15 274 for gland epithelium, and 1693 for hotspots), even with some misclassification of individual pixels these three classes approximate the average zinc content for gland epithelium, stroma, or hotspot reasonably well.

The protocol-dependent changes in zinc concentration in the gland epithelium, stroma, and hotspots followed the same overall trend as for the zinc content of the whole tissue (Fig. 6A). For the frozen and dried (FF and RTV) sections, the respective regions contained approximately the same amounts of zinc (glands ∼450 µg·g^–1^, stroma ∼340 µg·g^–1^, hotspots ∼850 µg·g^–1^). The stromal and glandular zinc content was reduced to about 40% after FFix and to about 16% after paraffin sealing (FFPS) of the respective frozen section concentrations. The hotspot zinc content remained stable after FFix and dropped to ∼50% after FFPS. Interestingly, the average ratio of all sections between the median zinc concentration of glands and stroma was 1.37 ± 0.09 (mean ± SD), thus the relative distribution between glands and stroma appears to be limited or not affected at all (Fig. 6B). At first glance, these results might appear devastating for other zinc imaging techniques that require tissue fixation and/or washing steps. However, our results indicate that even though zinc is quickly and strongly washed out of the tissue, the relative distribution between tissue types might be conserved (Fig. 6B). Nevertheless, even for qualitative analysis, a verification of this observed conservation is strongly recommended. Thus, any protocol using even few and brief washing steps should be carefully evaluated and if possible, discarded in favor of or at least verified by our optimal (RTV) protocol for LA–ICP–MSI when using fresh-frozen sections is disadvantageous or not possible.

The reduced washout effect of zinc in the hotspot areas suggests that zinc was present as salt or deposition with low solubility, such as zinc carbonate or zinc phosphate. The physiologically probable zinc citrate^19^ can be excluded due to its high solubility as can zinc phosphate due to the low phosphorous concentration in the hotspot regions (Fig. 3). Formalin fixation and subsequent brief washing steps did not affect zinc bound in the hotspot regions whereas the much numerous and longer washing during paraffination and dewaxing (FFPS) led to a clear zinc reduction. Of particular interest is the observation of exceptionally high zinc concentration in these prostatic deposits (zinc hotspots), which was accompanied by very low phosphorous in our analyses. This is in contrast to the described composition of such deposits, which were found to contain high levels of calcium phosphate.^20^ The origin, nature, and physiological/medical implications of these deposits are still a matter of debate^21^ and certainly require more research.

### Effects on other essential elements

Besides zinc, which was the target element in this study, the washout effect on other essential elements was also investigated. Phosphorous is present in every cell in compounds such as nucleotides and phospholipids with various solubility and size. Like previously described for zinc, drying at RT and vacuum packing did not influence the phosphorous intensity or distribution. Recovery rate for this protocol is 99.9% (Fig. 4, blue bars).

The other two protocols (FFix and FFPS) showed similar effects for phosphorous compared to zinc, resulting in recovery rates of 61.9 and 61.0%, respectively. For this element, fixation and washing steps already removed a major amount of soluble phosphorous and in this study paraffination and deparaffination did not enhance this effect further. A similar effect was also observed for iron. Iron is a part of proteins such as hemoglobin, myoglobin, or transferrin, all involved in iron transport and/or storage and as a co-factor in the form of iron–sulfur clusters present in many enzymes (e.g. aconitase, respiratory complexes I–III). Recovery for the RTV protocol (Fig. 4, green bars) was 96.2% and overlapped within the error bars with the fresh-frozen sections. Every additional sample preparation step tested in this paper led to major loss of iron up to recovery rates of 63.0 and 61.4% for fixed and sealed (FFix and FFPS) sections, respectively.

To summarize, our new protocol includes drying at RT and subsequent storage in vacuum. This strategy allows for more convenient storage and shipping under dry and stable temperature conditions (<25°C) mitigating the risk of damaging the tissue due to temperature fluctuation, especially during warmer seasons. The RTV protocol showed comparable results to fresh-frozen stored sections, which previously have been used for bioimaging techniques such as LA–ICP–MSI. For the prostate tissue used in this work, the recovery rates for the tested elements phosphorous, iron, and zinc were ≥96.2%, demonstrating an almost perfect measurement quality compared to the gold standard fresh-frozen storage condition. Although this is likely transferable to other tissue types, −80°C storage is still a gold standard and others may consider shipping both fresh frozen and RTV processed samples as backup so ensure that RTV is suitable for their experiments.

Further, the combination of simplicity and speed, is a clear advantage of the RTV protocol over closely related preparation protocols involving freeze drying. Freeze drying alone typically takes several hours and requires specialized equipment capable of maintaining high vacuum as compared to the 15 min drying step of the RTV protocol requiring only moderate vacuum achievable with household vacuum sealers. In addition, a recent study showed that freeze drying did not preserve the elemental composition of chicken liver samples analysed using LA–ICP–MS.^14^ This RTV protocol may also be applicable for other bioimaging approaches. It is likely applicable as a sample preparation technique prior to X-ray fluorescence microscopy-based elemental imaging, circumventing comparable challenges of target element washout caused by sample preparation.^22-24^ However, careful evaluation regarding oxidative states of target elements is required as oxidation states may not be fully preserved by the RTV protocol.^25^ Although other analytes such as metabolites, protein, RNA, and DNA are more sensitive to degradation, e.g. by autolysis, the dry vacuum condition in combination with moderate refrigeration should prevent or at least limit degradation. By removing water, the RTV protocol may have a comparable inhibitory effect on autolytic enzymes as other dehydrating tissue fixatives such as acetone or methanol, while simultaneously avoiding washout and delocalization of analytes. Several studies indicate that vacuum packing and cooling of whole tissue samples does conserve histologic integrity, DNA and protein, and to some degree also RNA and metabolites levels in a 72h timeframe.^26,27^ Nevertheless, temperature and storage time should be determined for the analytes and tissue type of interest. Finally, tissue integrity should be carefully assessed after drying. Depending on sectioning thickness and tissue composition, inevitable shrinkage during drying might lead to cracks that effect tissue morphology. We did not observe any drying-induced cracks in our samples large enough to be relevant for imaging at 15 µm resolution. However, formation of sub-micrometer cracks cannot be excluded. Thus, for cellular and sub-micrometer imaging the applicability of the RTV protocol might have limitations. Notably, as the carrier gas flow used during ablation will inevitably dry out any biological sample during measurement pre-drying under defined conditions might not be worse and could even prove beneficial.

## Conclusions

In this work, we presented a new tissue section preparation protocol for convenient handling and shipping for metal detection with LA–ICP–MSI between two different universities in Norway and Germany. Our new RTV protocol provides a simple and cost-efficient alternative for tissue exchange between international and interdisciplinary research projects without the disadvantage of washout effects observed in fixed samples. This protocol may also facilitate prolonged sample storage at higher temperatures and could probably be used for other bioimaging approaches. However, temperature and maximal storage time should be further determined for each tissue species and targeted analyte individually.

## Data Availability

The data and analysis code underlying this article are available in the Zenodo repository, at https://doi.org/10.5281/zenodo.6204296.
